# Association between Acculturation and Binge Drinking among Asian-Americans: Results from the California Health Interview Survey

**DOI:** 10.1155/2013/248196

**Published:** 2013-12-11

**Authors:** Monideepa B. Becerra, Patti Herring, Helen Hopp Marshak, Jim E. Banta

**Affiliations:** ^1^Department of Health Promotion and Education, School of Public Health, Loma Linda University, 24951 North Circle Drive, Loma Linda, CA 92350, USA; ^2^Department of Health Policy and Management, School of Public Health, Loma Linda University, 24951 North Circle Drive, Loma Linda, CA 92350, USA

## Abstract

*Objective*. Evaluate the association between acculturation and binge drinking among six Asian-American subgroups. *Methods*. A cross-sectional analysis of public access adult portion of 2007, 2009, and 2011/2012 California Health Interview Survey data was conducted. Univariate and multivariable logistic regression analyses were utilized with any binge drinking in the past year as the outcome variable and language spoken at home and time in USA as proxy measures of acculturation. *Results*. A total of 1,631 Asian-Americans (*N* = 665,195) were identified as binge drinkers. Binge drinking was positively associated with being first generation South Asian (OR = 3.05, 95% CI = 1.55, 5.98) and monolingual (English only) Vietnamese (OR = 3.00; 95% CI = 1.58, 5.70), especially among females. Other factors associated with increased binge drinking were being female (Chinese only), not being current married (South Asian only), and being an ever smoker (all subgroups except South Asians). *Conclusion*. First generation South Asians and linguistically acculturated Vietnamese, especially females, are at an increased risk of binge drinking. Future studies and preventive measures should address the cultural basis of such health risk behaviors among Asian-American adults.

## 1. Background

The Asian-American racial group is comprised of those having origins or immigrated from the Far East, Southeast East, or the Indian Subcontinent, thus consisting of a vast range of nationalities and reflective of a heterogeneous population. According to the US Census Bureau's 2010 Census Brief [1], a total of 10.2 million Asian-Americans (excluding those in combination with other races) were reported in 2000 and increased to 14.7 million by 2010, a 43.3% change. Of the Asian-American subgroups with at least one million responses were Chinese, Filipino, Asian-Indian, Vietnamese, Korean, and Japanese, with Asian-Indians experiencing the largest growth. Current estimates further report that by 2050, Asian-Americans are expected to comprise 9% of the entire US population, a rise of 4% compared to 2005 [2]. Such trends are indicative of an urgent need for research and health promotion measures to address the needs of a growing population.

The Asian-American population also varies in their socioeconomic status. For example, Asian-Indians are more likely to have a Bachelor's degree or higher compared to those who are Vietnamese. Similarly median household income can vary among Asian-Americans, ranging from $53,887 among Koreans to that of $90,528 among Asian-Indians [3]. Assessment of the National Health Interview Survey 2004–2006 further demonstrated that more than 75% of Japanese, Filipino, and Asian-Indian adults had incomes at or above 200% of the federal poverty level. On the other hand, Vietnamese, Koreans, and Chinese adults are twice as likely as Filipinos to at or below the poverty level [4], further demonstrating the heterogeneity among various Asian-American subgroups.

Similar to the aforementioned heterogeneous characteristics, cardiovascular disease (CVD) risk and behavioral patterns are also diverse among Asian-American subgroups. While the majority of current studies collapse the heterogeneous population into one, a few that have independently evaluated Asian-Americans demonstrated that certain sectors of the population, such as Asian-Indians and Filipinos, are at a greater risk of various CVDs than the general US population [4, 5]. For example, Barnes and colleagues [4] reported that Asian-Indian adults were twice as likely, as compared to Koreans to have ever been told to have heart disease.

Additionally, risk for hospitalization due to ischemic heart disease was significantly higher among Filipinos and South Asians, as compared to the referent group of Chinese adults [5]. Prevalence of type 2 diabetes mellitus, a risk factor for CVD, has also been reported to differ among Asian-Americans [6–9]. For example, diabetes prevalence was shown to be twice as high among Asian-Indians, as compared to Chinese and Japanese adults [4]. Using a nationally representative database, Ye and colleagues [9] also showed that, compared to Whites, Asian-Indians were 130% more likely to have diabetes.Despite such growing trends in the population and associated cardiovascular health outcomes, little research exists on elucidating the various health risk behaviors among the heterogeneous population, especially disaggregated by subgroups. This study examines the association between acculturation and binge drinking among six major Asian-American subgroups utilizing the California Health Interview Survey (CHIS), a population-based survey.

It is imperative to address current binge drinking behaviors as it is associated with significant negative health and socioeconomic consequences, including increased risk of cardiovascular diseases, motor vehicle accidents, violence, homicide, suicide, and loss of productivity [10–17]. Some researchers have suggested that moderate drinking could have cardioprotective effect [18], while others have highlighted that such potential cardioprotective role of moderate drinking could be overestimated due to lack of adequate adjustment for confounders [13]. Regardless of the debate whether light or moderate alcohol consumption can be a cardioprotective factor, several researchers have shown that at-risk drinking (binge or heavy) is associated with increased risk of CVD. Binge drinking is usually defined as 5 or more drinks for men and 4 or more drinks for women per occasion [19]. According to the Centers for Disease Control and Prevention, at least 15% of Americans report binge drinking in the past 30 days and currently 4 million binge drinking episodes occur each day [20]. Reducing binge drinking among US adults is one of the leading objectives of the Healthy People initiative [21] and given the increasing trends in binge drinking [22] and associated health outcomes, understanding the determinants of such behaviors is critical.

In recent years, a plethora of studies have highlighted the role of acculturation, the process by which immigrants adopt the views, attitudes, culture, and ways of the host nation [23, 24], in influencing various health behaviors, including alcohol consumption [25–28]. For example, Akins et al. [25] reported that acculturation was significantly associated with increased binge drinking with acculturated Hispanics reporting twice as much binge drinking as their nonacculturated counterparts. Similarly, high acculturation has been shown to increase drinking behavior among Hispanic women [26]. Zemore [28], in evaluating the 1995 National Alcohol Survey, demonstrated that acculturation was a significant predictor of various alcohol outcomes including drinking versus abstinence and average volume of drinks among drinkers.

The majority of such studies, however, have been conducted among the Hispanic population with limited research among Asian-Americans disaggregated by subgroups. Of the few studies among Asian-American subgroups, results remain limited in generalizability due to samples being limited to adolescent or college students [29–32]. Gomez and colleagues [33] assessed various health outcomes and behaviors among adult Asian-Americans by subgroup (Filipino, Chinese, and Japanese) including that of alcohol consumption. Results demonstrated that having a foreign language preference, thus low acculturation, was associated with lower odds of drinking. Despite highlighting the importance of acculturation, such results lack generalizability due to sample recruitment from a managed care setting and low sample size of some Asian-American subgroups, leading to collapsing such groups as “other Asian.” Given the heterogeneity of Asian-Americans it is critical to assess the determinants of such behaviors by distinct subgroup analysis to identify high-risk groups and thus the need for larger population-based studies, as further indicated by the authors themselves. Thus, utilizing CHIS, the largest state population-based survey in the nation, to evaluate the role of acculturation on binge drinking among adult Asian-Americans, disaggregated by subgroups (Chinese, Filipino, South Asian, Japanese, Korean, or Vietnamese), this study not only adds to the current limited body of literature but further highlights high-risk populations in need of health promotion measures.

## 2. Methods

### 2.1. Data Source

CHIS is a biennial population-based survey utilizing a random-digit-dial sample, including telephone and cellphones. Starting 2011, CHIS researchers released 2011/2012 combined data for public use. It is conducted in several languages, such as English, Spanish, Cantonese, Mandarin, Korean, and Vietnamese. Adults, who had a California address and telephone number, were at least 18 years of age or older, and completed ≥80% of the questionnaire, were included in the adult CHIS surveys. Those incarcerated, institutionalized, under 18 years of age, or residing in group quarters (dwelling where nine or more unrelated individuals lived together) were excluded. The mean ages of 2007, 2009, and 2001/2012 participants for CHIS were 53.8 years (standard deviation (SD) = 17.3), 55.7 years (SD = 17.3), and 55.1 years (SD = 18.0), respectively.

### 2.2. Sample

Data from adult respondents (18 years of age or older) who self-reported as one of the six Asian-American subgroups (Chinese, Filipino, South Asian, Japanese, Korean, or Vietnamese) in CHIS surveys were included in the study. This resulted in a total sample (*n*) of 12,839 participants for a population estimate (*N*) of 3,407,420 Asian-Americans in California.

### 2.3. Measures

The outcome variable for the study was any reported binge drinking (yes or no to binge drinking in the past 12 months), created from a CHIS-provided binge drinking variable. CHIS defined binge drinking as 5 or more drinks for men and 4 or more drinks for women per occasion. Due to lack of CHIS questionnaire on volume and types of drinks, further detailed analyses of other alcohol outcomes could not be included.

The exposure variable for this study was the latent construct of acculturation. Given that proxy measures of acculturation in current studies of the Asian-American population are varied, ranging from generation level [34] to country of birth [35], comparison across results remains difficult [36, 37]. To address such a limitation in the literature, this study included two proxies of acculturation: generation level and language spoken at home. Generation level was defined as zero (born outside USA), first (born in USA with both parents born outside USA), and second or more (born in USA and at least one parent born in USA). Language spoken at home was recoded from CHIS-provided variable to English only, English and another language, and a non-English language only. While CHIS assessed language of entertainment and language spoken with friends, such questions were also asked to a subset of the population. Moreover, variables such as language of interview and English language proficiency demonstrated strong multicolinearity with language spoken at home and thus only one was utilized. Similarly, variables such as citizenship status, country of birth, and years in USA had strong multicolinearity with generation level and thus only one was utilized.

Several studies have discussed the “alcohol income puzzle” where moderate and even heavy drinking have been associated with higher wages [38–40], a proxy for higher socioeconomic status. On the other hand, rates of alcohol abuse and associated outcomes have been associated with lower educational attainment [41, 42]. As a result, both education attainment (Bachelor's degree or more versus Associate degree or less) and poverty (at or above 200% federal poverty level versus below 200%) were utilized as covariates in the present study. Federal poverty level (FPL) takes into account both annual household income and size.

Our analyses further included smoking (ever versus never) as a potential covariate. The literature has consistently shown alcohol use and smoking to be strongly correlated, with some demonstrating tobacco use as a predictor of heavy alcohol use [43–45]. Moreover, in both men and women, poor health status has been associated with episodic heavy drinking [46] or frequent binge drinking [47]. Similarly, mental health status has been strongly associated with substance abuse, including alcohol [48, 49]. As a result, health status (poor versus good) was included in the study. Reporting at least one of the following was defined as poor health status: self-rated general health as poor, at least one chronic disease (diabetes, hypertension, heart disease, and congestive heart failure), Kessler 6-scale score of 13 or more indicating poor mental health [50, 51], and body mass index (BMI) of 23 kg/m^2^ or more, based on Asian-American BMI categories [52, 53].

Additionally, demographics, such as age, gender, and marital status, were further included. Finally, given that our study utilized several years of CHIS data, the year was included as a covariate to adjust for potential differences in sample size.

### 2.4. Statistical Analysis

Descriptive analyses were conducted to determine distribution of sociodemographics and other characteristics of each Asian-American subgroup in the study sample. Next, univariate analyses were performed using survey linear regression for continuous variable of age and Pearson's test, using design-based *F* values, for categorical variables, to assess if there were statistically significant differences between sociodemographics and other characteristics for Asian-Americans reporting binge drinking in the past 12 months. Multivariable logistic regression analyses were conducted, upon checking for assumptions, independently for each Asian-American subgroup with binge drinking as the dependent variable. Variables included in regression modeling were proxy measures of acculturation (language spoken at home and time in USA) along with covariates of age, gender, education level, poverty level, smoking behavior, health status, and survey year. A jackknife approach was utilized to adequately compute standard errors due to the survey's multistage complex sampling design, as in previous studies [54, 55]. Given that the literature has demonstrated acculturation to be gender specific [56, 57], interactions between each proxy measure of acculturation with gender, education, and poverty were further assessed using separate regression analyses after controlling for covariates. The criterion *α* for statistical significance was set at 0.05. Bonferroni adjustments were further conducted to reduce probability of type I error during each regression analysis by dividing 0.05 by the total number of independent variables. All statistical analyses were conducted using SAS 9.3 (SAS Institute, Inc., Cary, NC). The study was approved by Loma Linda University Institutional Review Board. 

## 3. Results

### 3.1. Univariate Analyses

Table 1 demonstrates the sample size, population estimates, sociodemographics, and other characteristics of binge drinkers compared to nonbinge drinkers for each Asian-American subgroup. The largest population estimates of binge drinkers were Filipinos (*N* = 246, 050) while the smallest group were Japanese (*N* = 42, 899). The mean age was significantly different among each subgroup (*P* < 0.0001), with the youngest binge drinkers being South Asians. Except for gender and marital status, significant differences were further noted for each characteristic across Asian-American subgroups.

### 3.2. Multivariable Analyses

Table 2 displays the results from multivariable logistic regression analyses with binge drinking as the outcome variable among six independent subgroups. Upon testing assumptions of logistic regression, age was nonlinear for several subgroups and thus a polynomial term was utilized. After adjusting for covariates, higher odds of binge drinking were associated with speaking only English at home among Vietnamese and being first generation among South Asians. South Asians reporting being currently unmarried were also more likely to binge drinks. Other characteristics associated with higher odds of binge drinking were being an ever smoker among all Asian-American subgroups except for South Asians. On the other hand, being female was significantly associated with lower binge drinking among Chinese subgroup. For all regression analyses presented in Table 2 Bonferroni adjustment resulted in criterion for *P* ≤ 0.004.

Significant interaction was obtained for gender and language spoken at home among Vietnamese subgroup only. While interactions of each acculturation proxy with poverty and education were assessed, no significant results (Bonferroni adjustment criterion of *P* ≤ 0.003) were obtained (data not shown). Independent regression analyses, upon adjusting for potential covariates (as in previous models), demonstrated that odds of binge drinking were significantly higher among bilingual Vietnamese females (adjusted odds ratio = 15.24; 95% confidence interval: 4.70, 49.45).

## 4. Discussion 

Currently, much of the empirical evidence on Asian-American health behaviors relies on collapsing the heterogeneous population as one group [30, 38]. By utilizing CHIS, a population-based survey, we were able to provide independent evaluation of six major Asian-American subgroups in California and further evaluate the association between acculturation and binge drinking behavior among such groups.

Our results highlight specific Asian-American subgroups, first generation South Asians, and monolingual (English only) Vietnamese, specifically females, as high-risk of binge drinking. The results from our study are partially consistent with previous studies on acculturation and cardiovascular behaviors. For example, Klonoff and Landrine [58] evaluated the role of acculturation on drinking behavior among African-Americans and demonstrated that abstainers were more likely to be traditional while the more acculturated ones were drinkers. In our study, a similar trend was observed among specific subgroups. Similarly, Zemore [28] also demonstrated that increased acculturation among Mexican-Americans was associated with drinking versus abstinence, though younger age was a higher predictor of frequency of drunkenness. While similar studies among Asian-American adults are limited, some have shown similar results. Gomez and colleagues [33] reported that among Asian-Americans lower odds of alcohol consumption were associated with a preference for foreign language, thus less acculturated. Similar to our results, the authors also noted that females were less likely to drink while not being currently married was associated with higher odds of drinking, though our results were specific to certain subgroups only.

Our study also showed that acculturation was not a significant predictor of binge drinking behavior among several Asian-American subgroups (Chinese, Filipinos, Japanese, and Koreans). While studies among adult Asian-Americans are limited, results from research among adolescent and college students may provide insight into such results. For example, Hahm et al. [29] noted that social factors, such as best friend's behavior, were a significant predictor of alcohol and tobacco use among Asian-American adolescents. On the other hand, Hendershot and colleagues [30] noted that acculturation was associated with decreased drinking behavior among college students. One of the potential explanations for such inconsistent results could be our disaggregation of Asian-Americans by subgroup thus leading to Simpson's paradox; where acculturation could be associated with drinking among all Asian-Americans collapsed as on group, but such a trend disappears upon subgroup analyses. Moreover, researchers have argued that acculturation can be either protective or negative based on an immigrant's native country [59]. For example, if a behavior, such as binge drinking, is likely to be high in the host nation, an acculturated immigrant is likely to increase such a behavior among immigration, resulting in the healthy immigrant paradox. Such a theory can partially explain the results of this study. For example, current epidemiologic data demonstrate that lifetime abstinence from alcohol is higher in Vietnam and India, immigrants of which nation are the majority of South Asians in the USA, as compared to the USA Thus, as expected immigrants from such nations are more likely to drink in the host nation. However, similar trends among Chinese and Filipinos were not observed, despite China and Philippines having higher abstinence rate than USA Due to significant association with smoking, prospective studies could potentially evaluate whether smoking behavior is a stronger predictor of alcohol consumption among such subgroups. Some studies have demonstrated that genetic variation, particularly the ALDH2∗2 allele involved in alcohol metabolism, is diverse among the Asian population, with higher rates noted among Chinese [60]. Partly, the differences in binge drinking rate could be attributed to this. However, researchers have further noted that ethnicity, particularly Korean, is associated with drinking, independent of ALDH2 status [61].

Consistent with the literature on the association between alcohol consumption and cigarette smoking [43, 44, 62, 63], our results also show a strong positive relationship between binge drinking and smoking among Asian-Americans, except for South Asians. The co-occurrence of two addictive behaviors should be a tremendous public health concern due to the negative health outcomes associated with both [64–66]. As a result, both alcohol abuse and tobacco prevention measures must address Asian-Americans with higher rates of both addictive behaviors in order to lower the associated morbidities and mortalities.

Additionally, Asian-Americans have often been considered a model minority due to socioeconomic achievements and low rates of alcohol consumption [67, 68]. Such a concept, however, has been consistently shown to be a myth based on recent empirical evidence demonstrating the heterogeneity among the population and specific high-risk groups [69–71]. Our study further adds to the literature by highlighting such high-risk Asian-American subgroups (Vietnamese and South Asians) and thus the need for binge drinking preventive measures. Based on our results, an average annual estimate of 665,195 Asian-Americans in California are binge drinkers, with the highest among Filipinos followed by the Chinese. Moreover, a recent study [72] utilizing CHIS 2005 data showed the heterogeneity in various health behaviors among Asian-American subgroups, though South Asians were excluded from such analysis. In our study, an annual population estimate of 83,462 South Asians reported being binge drinkers and given that our results show that first generation South Asians are at an increased risk of binge drinking, the need for further research among such a growing population [73] is imperative.

The results from this study should be interpreted with caution due to certain limitations. The cross-sectional design limits assessment of causality and demands further longitudinal studies. The self-reported data of CHIS is also susceptible to recall and social desirability biases. Moreover, due to lack of questionnaire in all Asian-American languages (especially South Asian languages) those with limited English proficiency are less likely to participate. The South Asian population in this study is also an aggregated group of various nationalities from the Indian Subcontinent. Generally South Asians have national origins from various countries, such as Bangladesh, Bhutan, India, Nepal, Pakistan, and Sri Lanka. As a result, while disaggregation of specific Asian-American subgroups is a critical component of this study, further analysis by specific South Asian groups is necessary. Additionally, other population based surveys, such as Behavioral Risk Factor Surveillance System, assess binge drinking in the past 30 days while starting 2007 the CHIS assessed binge drinking in the past 12 months, making such results difficult to be compared with other population-based surveys. Additionally, due to lack of questions on volume and types of drinks, more rigorous analysis of alcohol consumption behaviors among Asian-American subgroups could not be conducted.

The literature also suggests that current proxy measures of acculturation, including language and generation, may not adequately address all domains of acculturation [74]. Researchers have noted that language may only serve as an indirect measure of acculturation which further includes factors such as values and beliefs [75]. While lack of other domains of acculturation can be a limitation of the study, other researchers [76] have also acknowledged that other factors, such as values, customs, are often embedded in language and thus such a measure can adequately serve as a proxy for acculturation in population-based studies. Since CHIS lacks assessment of such domains, the acculturation proxies utilized in this study may not provide a comprehensive assessment of acculturation among Asian-Americans. Finally, due to the limitation of state samples, results from this study may not be generalizable to Asian-Americans residing outside of California.

Despite such limitations, the present study provides a significant contribution to the literature. CHIS is a population-based survey utilizing random-digit-dial system, thus reducing selection bias. The statistical adjustments in the study, including sample weights, further minimize selection biases and make results generalizable to Asian-Americans in California. Given that, based on Census 2010 [1], California reports 4,861,007 Asian-alone groups, the highest among all other states and Puerto Rico, CHIS provides an ideal scope of evaluation of health behaviors and determinants of such outcomes among the population.

## 5. Conclusion 

The Healthy People 2020 initiative provides science-based, 10-year national benchmarks for improving the health and quality of life for all Americans. An integral component of this national initiative is to improve the nation's cardiovascular health by 20% [77]. This study provides critical empirical evidence of binge drinking, a cardiovascular health risk behavior, among Asian-American subgroups in California. The positive association between acculturation and binge drinking among specific Asian-American subgroups, as demonstrated in this study, can further provide health educators the foundations for setting preventive strategies. Some studies have suggested that traditional view towards alcohol could be a factor associated with drinking behavior. For example, a study [78] noted that while Pakistani young adults (in the UK) maintained similar views against alcohol as their parents', they further recognized that increased levels of drinking among their population are often unrecognized by the community leading to as the authors described “generational dislocation,” a potential phenomenon noted in our study with higher binge drinking among first generation South Asians. Moreover, given the importance of acculturation highlighted in this study, further evaluation of the role of such a latent construct on other cardiovascular health behaviors, such as diet, physical activity, and smoking, among Asian-American subgroups, is warranted.

## Figures and Tables

**Table:** (a)

	Chinese		Filipino		South Asian	
	Binge	Nonbinge	Binge	Nonbinge	Binge	Nonbinge
Total sample size (*n*)	311	3265	310	1328	163	1189
Average annual population estimate of binge drinkers (*N*)	130,357	921,453	246,050	654,662	83,462	384,216
Mean age (95% CI)	32.89 (31.12, 34.66)	45.70 (44.84, 46.57)	34.24 (32.20, 36.27)	46.40 (45.27, 47.54)	32.17 (29.94, 34.41)	39.40 (38.44, 40.37)
Female (%)	32.91	56.26	38.98	60.54	32.18	46.09
Currently married (%)	36.76	63.09	42.42	62.00	36.74	74.07
Bachelor's degree or higher (%)	62.11	55.25	39.53	55.30	81.40	76.01
Less than 200% FPL (%)	21.14	31.71	18.68	26.46	11.38	18.07
Language spoken at home (%)						
Non-English only	38.99	44.54	3.71	13.00	5.69	14.37
English and another	42.49	39.46	47.90	52.60	65.16	71.47
English only	18.51	16.00	48.39	34.40	29.15	14.15
Generation level (%)						
Zero	60.92	77.24	49.07	75.44	57.97	88.72
First	31.58	17.31	36.97	17.96	41.17	9.83
Second or more	7.49	5.45	13.96	6.60	0.86	1.44
Ever smoker (%)	30.38	16.38	48.58	23.94	23.14	12.60
Poor health status (%)	36.99	44.03	17.04	24.17	40.59	37.37

**Table:** (b)

	Japanese		Korean		Vietnamese		*P* value	
	Binge	Nonbinge	Binge	Nonbinge	Binge	Nonbinge	Binge	Nonbinge
Total sample size (*n*)	151	1024	391	1907	305	2495		
Average annual population estimate of binge drinkers (*N*)	42,899	179,492	90,729	249,920	71,700	352,484		
Mean age (95% CI)	41.51 (37.94, 45.09)	57.26 (55.45, 59.08)	36.46 (33.66, 39.26)	46.07 (44.39, 47.75)	32.91 (29.55, 36.27)	45.01 (43.80, 46.22)	0.0002	<.0001
Female (%)	49.98	60.42	51.25	68.95	38.21	53.34	0.1578	<.0001
Currently married (%)	48.11	65.75	47.76	59.50	33.95	64.60	0.3804	.0005
Bachelor's degree or higher (%)	59.77	51.64	55.55	57.33	43.95	28.93	<.0001	<.0001
Less than 200% FPL (%)	16.02	13.29	22.10	35.90	34.86	56.18	0.0205	<.0001
Language spoken at home (%)							<.0001	<.0001
Non-English only	6.54	3.83	36.60	52.06	16.75	52.35		
English and another	28.98	25.12	46.36	37.24	73.81	40.40		
English only	64.48	71.05	17.04	10.70	9.43	7.25		
Generation level (%)							<.0001	<.0001
Zero	32.58	25.72	67.21	81.13	53.01	90.04		
First	7.77	20.28	31.59	17.00	46.67	9.54		
Second or more	59.65	53.99	1.20	1.87	0.32	0.42		
Ever smoker (%)	47.31	30.47	50.74	27.39	52.94	24.36	<.0001	<.0001
Poor health status (%)	33.49	23.98	43.89	42.83	51.48	43.33	<.0001	<.0001

CI: confidence interval; FPL: federal poverty level.

**Table 2:** Multivariable logistic regression odds ratio (and 95% confidence interval) for binge drinking among adult Asian-Americans.

	Chinese	Filipino	South Asian	Japanese	Korean	Vietnamese
Acculturation						
Language spoken at home						
Non-English only (ref.)						
English and another	0.98 (0.55, 1.74)	1.99 (0.83, 4.77)	2.12 (0.85, 5.33)	0.56 (0.19, 1.61)	1.32 (0.58, 2.99)	1.62 (0.51, 5.22)
English only	0.83 (0.52, 1.33)	2.91 (1.21, 6.96)	1.54 (0.72, 3.27)	0.52 (0.17, 1.59)	0.98 (0.59, 1.63)	3.00 (1.58, 5.70)*
Generation level						
Zero (ref.)						
First	0.97 (0.59, 1.60)	1.50 (0.88, 2.55)	3.05 (1.55, 5.98)*	0.35 (0.08, 1.57)	1.70 (0.73, 3.96)	3.34 (0.67, 16.65)
Second or more	1.50 (0.74, 3.05)	2.25 (0.76, 6.68)	0.72 (0.11, 4.60)	0.57 (0.28, 1.16)	0.38 (0.04, 3.64)	0.92 (0.10, 8.68)
Gender						
Male (ref.)						
Female	0.49 (0.33, 0.74)*	0.75 (0.47, 1.20)	0.75 (0.40, 1.41)	0.66 (0.38, 1.16)	0.49 (0.27, 0.90)	0.86 (0.40, 1.84)
Marital status						
Currently married (ref.)						
Not currently married	1.44 (0.83, 2.50)	1.55 (0.96, 2.51)	3.99 (1.95, 8.16)*	2.21 (1.16, 4.22)	1.19 (0.57, 2.51)	1.79 (0.97, 3.30)
Education level						
Associate degree or less (ref.)						
Bachelor's degree or higher	1.46 (0.92, 2.33)	0.70 (0.46, 1.08)	2.05 (0.88, 4.81)	1.64 (0.90, 3.00)	0.65 (0.38, 1.11)	1.94 (1.04, 3.63)
Poverty level						
<200% FPL (ref.)						
≥200% FPL	1.41 (0.82, 2.42)	1.87 (1.14, 3.09)	1.51 (0.69, 3.29)	1.20 (0.50, 2.91)	1.66 (0.89, 3.08)	1.13 (0.60, 2.13)
Health status						
Good (ref.)						
Poor	1.62 (1.00, 2.65)	1.83 (0.99, 3.38)	1.53 (0.90, 2.59)	0.63 (0.32, 1.23)	1.21 (0.70, 2.08)	1.22 (0.70, 2.13)
Smoking status						
Never smoker (ref.)						
Ever smoker	2.86 (1.89, 4.33)*	3.21 (2.05, 5.05)*	1.89 (0.95, 3.75)	4.26 (2.38, 7.64)*	2.70 (1.44, 5.07)*	8.43 (3.73, 19.06)*
Survey year						
2007 (ref.)						
2009	1.77 (1.00, 3.14)	0.96 (0.59, 1.58)	1.31 (0.63, 2.70)	1.16 (0.60, 2.25)	1.16 (0.63, 2.14)	1.06 (0.48, 2.34)
2011/2012	2.13 (1.39, 3.25)*	1.04 (0.71, 1.54)	0.92 (0.47, 1.80)	3.04 (1.52, 6.07)*	0.97 (0.51, 1.83)	1.28 (0.57, 2.87)

FPL: federal poverty level.

*Bonferroni adjustment criterion of *P* < 0.004.
